# Penetration and distribution of gadolinium-based contrast agents into the cerebrospinal fluid in healthy rats: a potential pathway of entry into the brain tissue

**DOI:** 10.1007/s00330-016-4654-2

**Published:** 2016-11-10

**Authors:** Gregor Jost, Thomas Frenzel, Jessica Lohrke, Diana Constanze Lenhard, Shinji Naganawa, Hubertus Pietsch

**Affiliations:** 1 MR and CT Contrast Media Research, Bayer Pharma AG, Muellerstrasse 178, 13353 Berlin, Germany; 2 Institute of Vegetative Physiology, Charité, Berlin, Germany; 3 Department of Radiology, Nagoya University Graduate School of Medicine, Nagoya, Japan

**Keywords:** Magnetic resonance imaging, Gadolinium, Contrast media, Cerebrospinal fluid, Brain

## Abstract

**Objective:**

Signal hyperintensity on unenhanced MRI in certain brain regions has been reported after multiple administrations of some, but not all, gadolinium-based contrast agents (GBCAs). One potential initial pathway of GBCA entry into the brain, infiltration from blood into the cerebrospinal fluid (CSF), was systematically evaluated in this preclinical study.

**Methods:**

GBCA infiltration and distribution in the CSF were investigated in healthy rats using repeated fluid-attenuated MRI up to 4 h after high-dose (1.8 mmol/kg) administration of six marketed and one experimental GBCA. Additionally, gadolinium measurements in CSF, blood and brain tissue samples (after 24 h) were performed using inductively coupled plasma mass spectrometry.

**Results:**

Enhanced MRI signals in the CSF spaces with similar distribution kinetics were observed for all GBCAs. No substantial differences in the gadolinium concentrations among the marketed GBCAs were found in the CSF, blood or brain tissue. After 4.5 h, the concentration in the CSF was clearly higher than in blood but was almost completely cleared and lower than the brain tissue concentration after 24 h.

**Conclusions:**

In contrast to the brain signal hyperintensities, no differences in penetration and distribution into the CSF of healthy rats exist among the marketed GBCAs.

***Key Points*:**

• *Gadolinium-based contrast agents can cross the blood-CSF barrier*.

• *Fluid-attenuated MRI shows GBCA distribution with CSF flow*.

• *GBCA structure and physicochemical properties do not impact CSF penetration and distribution*.

• *GBCA clearance from CSF was almost complete within 24 h in rats*.

• *CSF is a potential pathway of GBCA entry into the brain*.

## Introduction

Gadolinium-based contrast agents (GBCAs) are frequently used in MRI examinations and are generally considered to have an excellent safety profile [1]. Following intravenous injection, GBCAs distribute in the blood and the extravascular-extracellular space; however, to the common knowledge these agents cannot penetrate the intact blood-brain barrier (BBB) [2]. Therefore, CNS imaging – i.e. the possibility to enhance areas with a disrupted BBB – is a major indication for contrast-enhanced MRI [3]. However, after multiple administrations of GBCAs, increased signal intensities (SIs) were recently reported on unenhanced T1-weighted MRIs in certain brain regions, mainly in patients with multiple sclerosis or neoplastic diseases [4–12]. A correlation between these signal hyperintensities in the dentate nucleus (DN) and globus pallidus (GP) and the number of contrast-enhanced MRI examinations was first described by Kanda et al. in 2014 [4]. Subsequent autopsy studies verified the presence of gadolinium in enhanced brain structures and suggest a correlation between the gadolinium present in the DN and GP and T1-weighted MRI SI increase [13, 14]. The SI increase seems to be primarily associated with the repeated use of the multi-purpose linear GBCAs gadodiamide, gadopentetate dimeglumine and gadobenate dimeglumine [4–12, 15, 16]. For gadopentetate dimeglumine a significant change of T1 was also confirmed by MR-relaxometry [17]. In the brain of decedents gadolinium was detected after administration of linear and macrocyclic GBCAs [18]. However, no visible SI increase has been observed for the macrocyclic GBCAs gadobutrol, gadoterate meglumine and gadoteridol [7, 11, 12, 19–21]. Stojanov et al. reported that gadobutrol causes signal enhancement of the DN and GP [22]. However, the design of this study was critically discussed and does not convincingly support this conclusion [23–25].

In order to study this more systematically and especially under controlled and reproducible conditions, animal models were established and confirmed prospectively the brain signal hyperintensities seen in the retrospective patient studies [26–28]. The animals showed elevated SIs in the cerebellar nuclei after multiple administrations of linear but not after receiving macrocyclic GBCAs [26–28]. It is important to note that the animals were healthy, without underlying diseases that may affect BBB integrity and function.

The mechanism of GBCA accumulation in the brain is unknown and raises several questions. This study focuses on the initial pathway of GBCA entry into the brain. In the brain, two barrier systems exist, the BBB and the blood-cerebrospinal fluid (CSF) barrier [29]. Regarding brain signal hyperintensities in clinical studies, the status of the patients’ BBB integrity and function is either unknown or can be most likely characterized as a local BBB disruption due to disease processes. However, the underlying mechanism that allows GBCA infiltration into the brain, in particular in areas that are anatomically distant from a disease process, is not known. In addition to a disrupted BBB, GBCA penetration from the blood into the CSF represents another potential pathway for GBCA entry into the brain. Indeed, CSF enhancement in the internal auditory canal has been described after intravenous GBCA administration in patients with Ménière’s disease [30, 31]. However, little knowledge exists about the impact of the physicochemical properties and chemical structure of GBCAs on the distribution and intensity of the enhancement. The impact of time on signal enhancement in various cranial fluid spaces after the administration of gadoteridol was evaluated in healthy volunteers using heavily T2-weighted fluid-attenuated inversion recovery (FLAIR) imaging [32]. This dedicated sequence is highly sensitive to detect low GBCA concentrations [30]. In a preclinical study, CSF signal enhancement on FLAIR images was initially observed for all GBCAs investigated, suggesting that all GBCAs could pass the blood-CSF barrier in rats to a certain, but not yet quantified, extent [27].

The aim of this preclinical study was to evaluate the penetration of GBCAs from the blood into the CSF and the distribution kinetics within different CSF cavities in a systematic manner. Therefore, GBCAs with linear and macrocyclic structures and different physicochemical properties (ionic, non-ionic and protein-binding) were evaluated by FLAIR MRI up to 4 h post injection (p.i.). In addition to the marketed GBCAs, the experimental macromolecular agent gadomer was used to assess the effect of molecular size. CSF and blood samples were obtained at 4.5 and 24 h after GBCA administration for inductively coupled plasma-mass spectrometry (ICP-MS)-based gadolinium quantification. Additionally, samples from the cerebellum and pons were analysed to determine gadolinium concentration 24 h p.i.

## Material and methods

### Animals

One hundred and two healthy Han-Wistar rats (Crl:WI; males; 275–325 g) were obtained from Charles River (Sulzfeld, Germany). The animals were kept under standard laboratory conditions and standard rat chow and water were provided ad libitum. The animals were handled and treated according to German animal regulations. The MRI and CSF sampling were performed under anaesthesia with 1.5 % isoflurane (Baxter GmbH, Unterschleißheim, Germany).

### Study setup

**Fig. 1:**
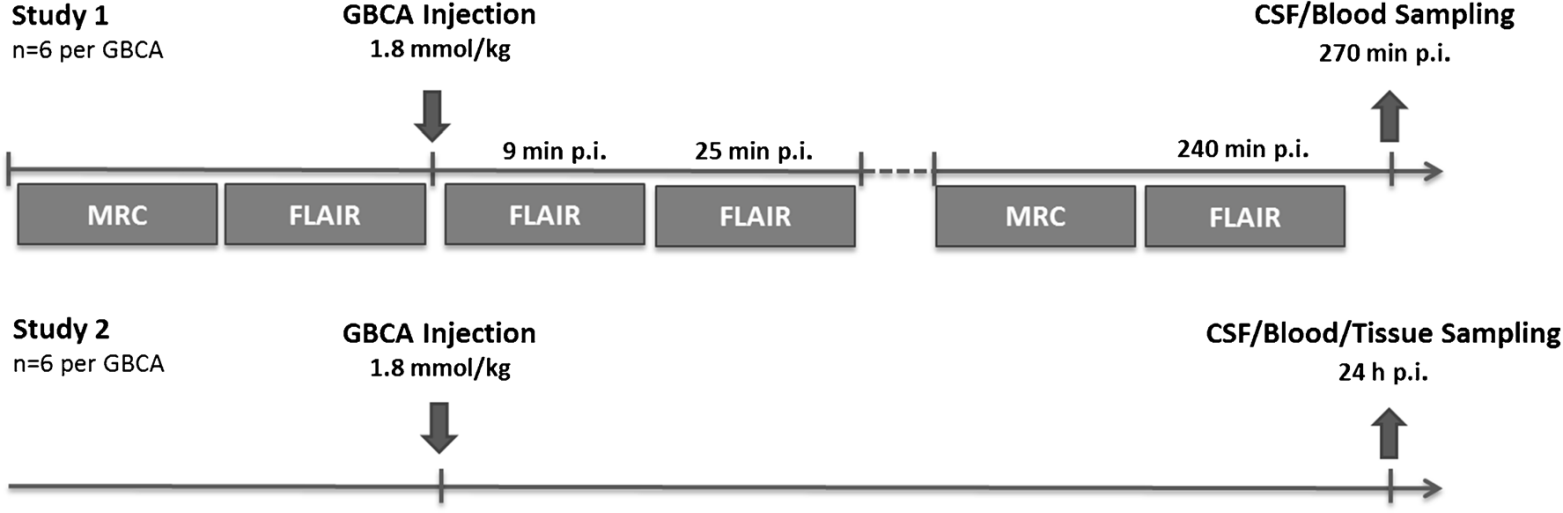
Experimental setup. The study was performed in two parts, each with six animals per experimental group. Part 1 (upper row) included MR-cisternography (MRC) and fluid-attenuated imaging (FLAIR) of the rat brain before and up to 240 min after injection of GBCA. After 270 min a blood and CSF sampling was performed. In part 2 (lower row) blood, CSF and samples from the cerebellum and pons were obtained 24 h after injection. No MRI was done in this part of the study. *p.i.* post injection

The study was performed in two parts: First, an MRI-based evaluation of GBCA infiltration and distribution within the CSF up to 4 h after the administration, and second, the quantification of CSF, blood, cerebellum and pons gadolinium concentration 24 h p.i. (Fig. 1). Ninety-six rats were randomly divided into a control and seven GBCA groups (n = 12 each), and additional six rats were used to monitor quantitative changes of SI after a necessary service of the MRI system. For the two study parts, each group was divided into two subgroups (n = 6 each). The first subgroup underwent MR-cisternography (MRC) and FLAIR MRI up to 4 h after GBCA administration. Subsequently, a CSF and blood sample was obtained for ICP-MS-based gadolinium quantification. Animals from the second subgroup underwent CSF and blood sampling 24 h after GBCA injection and samples from the cerebellum and pons were also taken. The following GBCAs were investigated: gadopentetate dimeglumine (Magnevist, Bayer Vital GmbH, Leverkusen, Germany), gadodiamide (Omniscan, GE Healthcare Buchler GmbH, Braunschweig, Germany), gadobenate dimeglumine (MultiHance, Bracco Imaging Deutschland GmbH, Konstanz, Germany), gadobutrol (Gadovist, Bayer Vital GmbH), gadoterate meglumine (Dotarem, Guerbet GmbH, Sulzbach/Taunus, Germany) and gadoteridol (ProHance, Bracco Imaging Deutschland GmbH). The experimental GBCA gadomer (Bayer Pharma AG, Berlin, Germany) was used to assess the effect of molecular size. Gadomer is a dendritic gadolinium complex with a molecular weight of 17 kDa that distributes almost exclusively within the intravascular compartment [33]. All agents were applied intravenously at a dose of 1.8 mmol Gd/kg body weight. This dose approximates three times the human standard dose based upon body surface area normalization between rats and humans [34]. Saline administered at identical volume was used in the control group. During the experiments, servicing of the MR scanner was necessary. The images of the experimental gadomer and gadoteridol groups that underwent MRI after the service showed altered SI levels. Therefore, the additional gadobutrol group (n = 6) was added to ensure comparability between the first and second set of MRI measurements.

### MRI protocol

MR imaging was performed using clinical 1.5-T MRI (Avanto, Siemens Healthcare GmbH, Erlangen, Germany) and a dedicated two-channel rat head coil (Rapid Biomedical GmbH, Rimpar, Germany). For the evaluation of the fluid space, T2-weighted MRC for anatomical references of the CSF space was used. For MRC, a variable flip angle 3D-TSE sequence (TR = 4,400 ms, TE = 553 ms) with an initial refocusing flip angle of 180° (decreased to 120° for the refocusing echo train) and a turbo factor of 79 were used. The spatial resolution was 0.3 × 0.3 × 0.6 mm (field of view (FOV) = 100 × 48 mm; 30 transversal slices). MRC was followed by pre- and post-contrast heavily T2-weighted FLAIR sequence with identical parameters. However, a non-selective inversion recovery pulse with an inversion time of 2,250 ms was included, and TR was extended to 9,000 ms. The first FLAIR sequence was started 1 min after GBCA injection immediately followed by a second scan. The middle of the scan time (16:14 min) was set as the time point for the temporal evaluation (9 min and 25 min p.i., respectively). Another MRC and FLAIR measurement was performed 4 h p.i.

### Gadolinium quantification

For CSF sampling, the anaesthetized animals were positioned in a stereotactic frame. The head was flexed, and a small incision was made inferior to the occiput to expose the dura mater of the cisterna magna. A catheter capillary tube was inserted into the cisterna magna, and the CSF was collected into the tube. The animals were euthanised by exsanguination, and a blood sample was collected. In the second part of the study, the brain was removed and dissected to sample the cerebellum and pons. The gadolinium concentrations of the CSF, blood, cerebellum and pons were measured by ICP-MS (Agilent 7500a, Waldbronn, Germany).

### Image evaluation

**Fig. 2:**
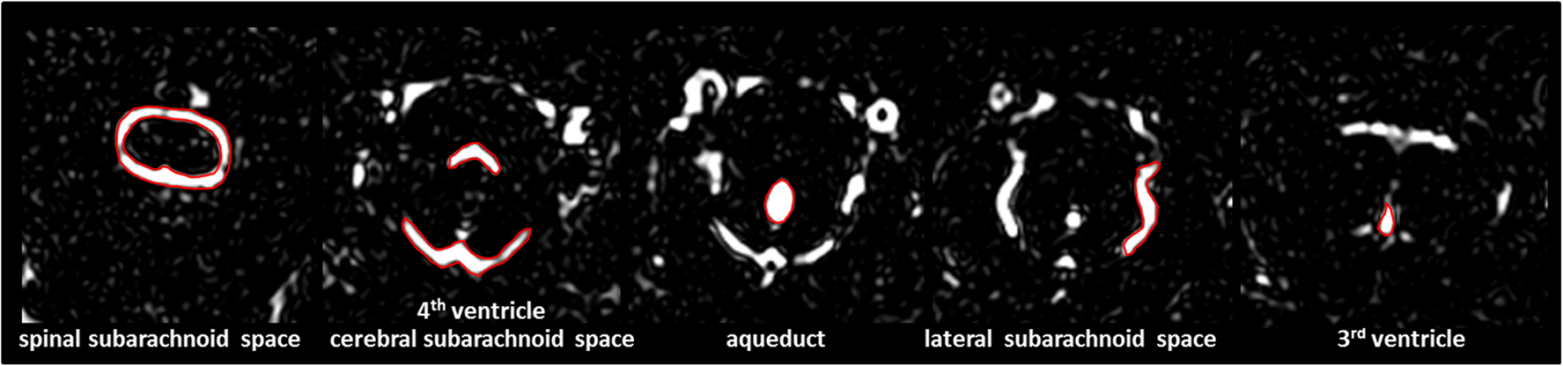
Examples for region of interest (ROI) placement. For the quantitative analysis ROIs were placed around different CSF spaces using the MR-cisternography (MRC) images for anatomical reference

Image analysis was performed by two experienced readers who were blinded to the experimental groups. In the MRC images, manually defined regions of interest (ROIs) were drawn in two adjacent slices around the third and fourth ventricles, the aqueduct and in the subarachnoid space at different anatomical locations (cerebral, spinal and lateral). Examples of the ROI placement are shown in Fig. 2. The ROI positions were copied to the FLAIR images to evaluate the contrast enhancement before and after GBCA administration.

## Results

### CSF enhancement

**Fig. 3:**
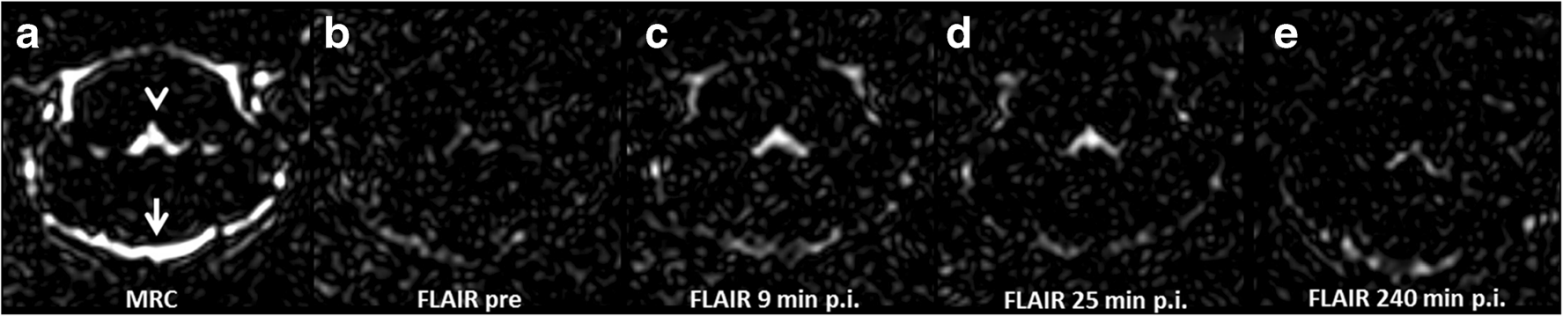
Representative images. The CSF spaces were visualised by MR-cisternography (MRC), for example the fourth ventricle (arrowhead) and the subarachnoid space (arrow) (**a**). In the fluid-attenuated (FLAIR) images before GBCA injection the respective CSF signal is almost completely attenuated (**b**). After GBCA administration a clear signal enhancement of the CSF spaces was found in the FLAIR images up to 240 min post injection (p.i.) (**c**–**e**)

Signal enhancement of the CSF spaces was observed in all FLAIR images after GBCA administration. Figure 3 shows representative images of the brain at the level of the fourth ventricle. The cavity fluid spaces are visualized by MRC to determine the anatomical location and are almost completely attenuated in the baseline FLAIR scan (Fig. 3b). A clearly visible signal enhancement indicating the presence of GBCA in the fluid spaces was demonstrated by FLAIR imaging after 9 min, 25 min and 240 min p.i. (Fig. 3c–e). The FLAIR imaging throughout the brain depicted GBCA-induced signal enhancement in all ventricles and the subarachnoid space.

**Fig. 4:**
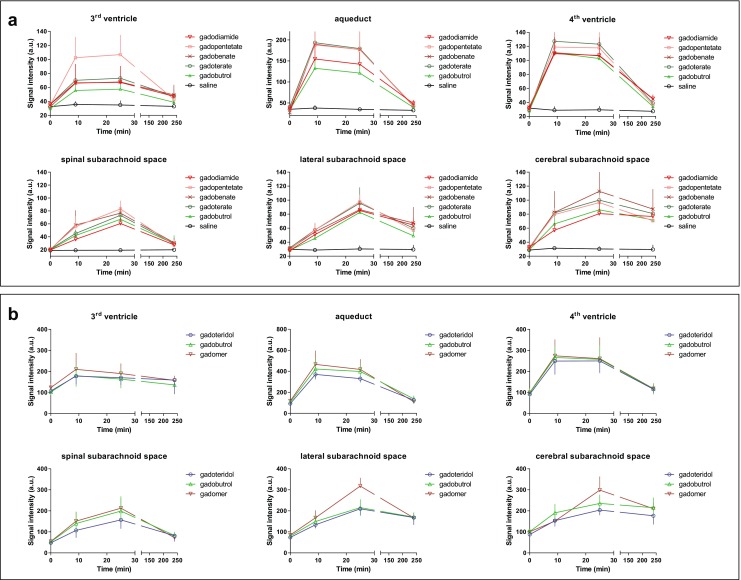
Quantitative analysis of CSF signal intensity (SI). The analysis of the SI on fluid-attenuated (FLAIR) images over time in different CSF spaces was divided into two parts (**a** and **b**) because the SI for the baseline scans (t = 0 min) was significantly higher after servicing of the MRI scanner. However, each analysis includes a gadobutrol group to ensure comparability. gadopentetate = gadopentetate dimeglumine; gadobenate = gadobenate dimeglumine; gadoterate = gadoterate meglumine; error bars represent standard deviation

For quantitative image analysis ROIs were placed at different CSF spaces to evaluate the distribution pattern and kinetics of the GBCAs over 240 min (Fig. 4). Gadoteridol, gadomer and the second gadobutrol group were diagrammed separately (Fig. 4b) due to a shift in the SI levels after servicing of the MR scanner. Comparing the two gadobutrol groups (Fig. 4a and b), the different signal levels were obvious.

CSF signal enhancement was detected for all GBCAs at comparable levels. A rapid signal enhancement was found immediately after administration in the inner CSF cavities (third and fourth ventricle, aqueduct), which was followed by successively declining CSF signals. After 240 min the SI reached almost baseline level. By comparison, the CSF signal in the subarachnoid space (spinal cord, lateral and cerebral location) increased at a slower rate with a peak at 25 min p.i. Subsequently, decreasing SIs were observed until 240 min p.i., declining more rapidly and rigorously at the level of the spinal cord than on the lateral and cerebral level.

### Gadolinium quantification

**Fig. 5:**
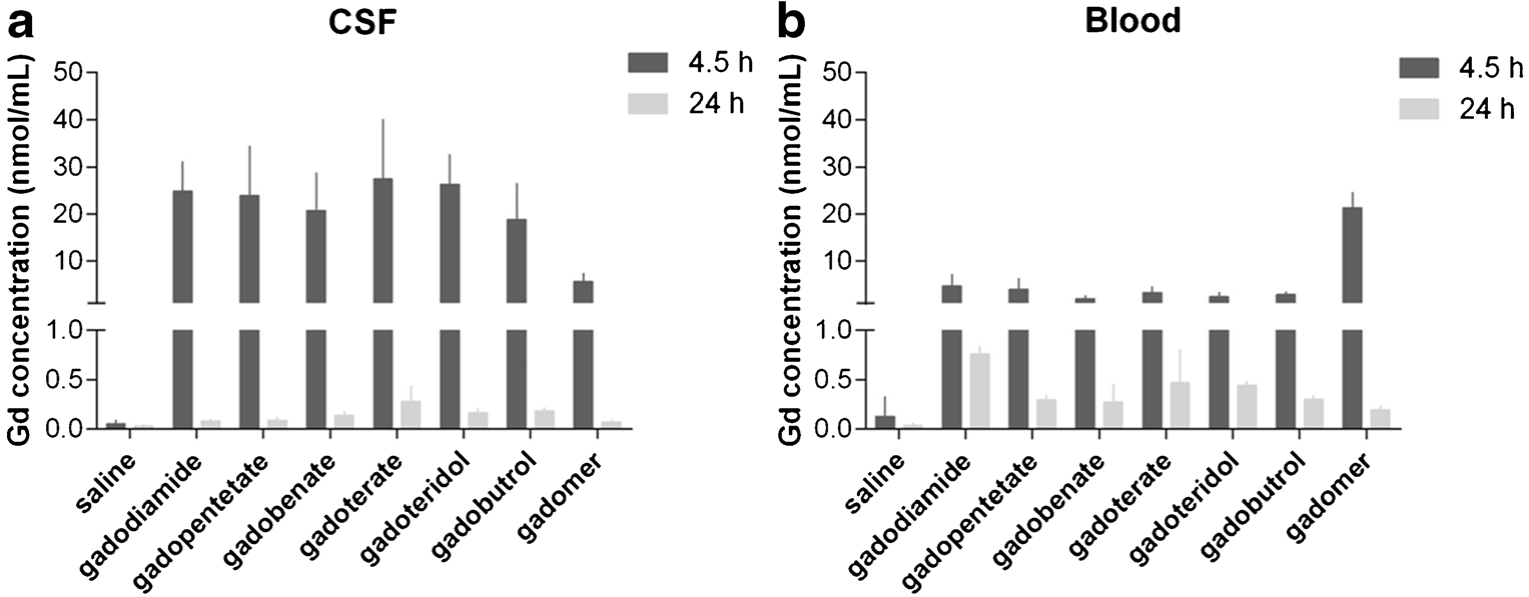
Gadolinium (Gd) concentrations in CSF and blood. The gadolinium concentration determined in the CSF (**b**) and blood (**b**) samples obtained at 4.5 h and 24 h, respectively. gadopentetate = gadopentetate dimeglumine; gadobenate = gadobenate dimeglumine; gadoterate = gadoterate meglumine; error bars represent standard deviation

Analytical determinations of the gadolinium concentrations in the CSF and blood were conducted 4.5 and 24 h post GBCA injection by ICP-MS (Fig. 5). At 4.5 h, the CSF gadolinium concentrations for the marketed GBCAs were in the range of 18.8 ± 7.7 nmol/ml (gadobutrol) to 27.4 ± 12.7 nmol/ml (gadoterate meglumine). For gadomer, a clearly lower concentration of 5.5 ± 7.7 nmol/ml CSF was detected. After 24 h, the GBCAs were almost completely cleared from the CSF, and the concentrations of the marketed GBCAs ranged from 0.08 ± 0.02 nmol/ml (gadodiamide) to 0.28 ± 0.16 nmol/ml (gadoterate meglumine). For gadomer, a gadolinium concentration of 0.07 ± 0.02 nmol/ml was detected. Compared with blood, higher concentrations were found in the CSF for all marketed GBCAs at 4.5 h. However, after 24 h all concentrations in the CSF were below the blood gadolinium levels. In the blood, comparable gadolinium concentrations were observed 4.5 h after administration of the marketed GBCAs. The concentration ranged from 2.0 ± 0.7 nmol/ml (gadobenate dimeglumine) to 4.7 ± 2.4 nmol/ml (gadodiamide). After 24 h, the remaining concentrations were in the range between 0.27 ± 0.19 nmol/ml (gadobenate dimeglumine) and 0.76 ± 0.08 nmol/ml (gadodiamide).

**Fig. 6:**
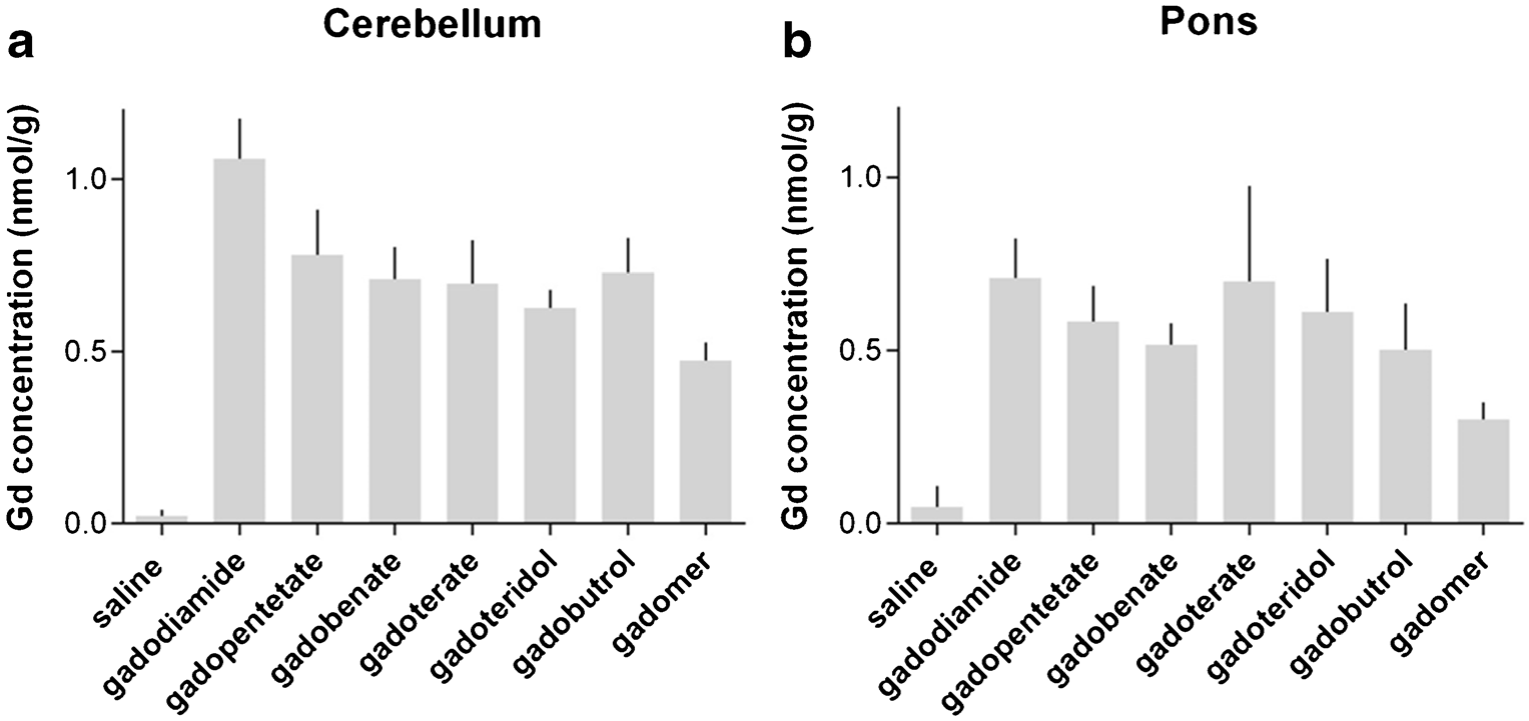
Gadolinium (Gd) concentrations in the cerebellum and pons. The gadolinium concentration per gram tissue determined in the cerebellum (**a**) and pons (**b**) 24 h after administration. gadopentetate = gadopentetate dimeglumine; gadobenate = gadobenate dimeglumine; gadoterate = gadoterate meglumine; error bars represent standard deviation

The gadolinium concentrations measured in the cerebellum and pons obtained 24 h p.i. were higher than those in the CSF and blood. In the cerebellum, the concentrations for the marketed GBCAs were in the range of 0.63 ± 0.05 nmol/g (gadoteridol) to 1.06 ± 0.05 nmol/g (gadodiamide). In the pons, slightly lower gadolinium concentrations were detected. In both structures analysed, the lowest concentrations were found for gadomer (Fig. 6).

## Discussion

In this preclinical rat study, the amount and kinetics of GBCA infiltration and distribution in the CSF were investigated by FLAIR MRI up to 4 h p.i. Additionally, gadolinium measurements in the CSF, blood cerebellum and pons were performed by ICP-MS. For this purpose, six marketed GBCAs with different structural and physicochemical properties and one experimental agent with significantly larger molecular size were evaluated.

Comparable CSF signal enhancements on FLAIR images were observed for all GBCAs independent of their chemical structure or physicochemical properties such as the ionicity or ability to bind partially to proteins. The kinetics of signal enhancement differs between the inner CSF cavities (ventricles and aqueduct) and subarachnoid space. The faster signal increase in the inner cavities demonstrates that the primary location of GBCA infiltration is most likely the choroid plexus located in the ventricles. The fenestrated capillaries of the choroid plexus are relatively permeable to smaller substances, such as GBCAs, which can pass into the choroid plexus interstitium. The choroid plexus continuously secretes CSF, and the choroid plexus epithelium forms the blood-CSF barrier. Importantly, the blood-CSF barrier is known to be physiologically more leaky than the BBB [3, 35].

This study demonstrated that all marketed GBCAs cross the blood-CSF barrier to an almost identical extent. However, the ability to cross this barrier seems to depend on the molecular size as demonstrated by the considerably lower CSF gadolinium concentration for the experimental gadomer which is significantly larger (17 kDa) than the marketed GBCAs (<1 kDa). In contrast to the analytical gadolinium quantification, the reduction was not observed with FLAIR MRI as the r1 relaxivity of gadomer is about a factor of five higher than that of the other GBCAs [36]. After penetrating the blood-CSF barrier, further GBCA distribution within the CSF is driven by diffusion, convection and CSF flow that are directed through the ventricles to the subarachnoid space of the cortex and spinal cord. The delayed MRI signal increase observed in the subarachnoid space represents this distribution process.

For the marketed GBCAs, the averaged CSF gadolinium concentrations are about a factor of 7.4 higher than the respective blood concentrations at 4.5 h p.i. However, after 24 h GBCAs are almost completely cleared from the CSF, and the respective gadolinium concentrations are much lower than those in the blood. This is in contrast to gadolinium concentrations in the cerebellum and pons that are higher than those in the CSF and blood at 24 h p.i. This demonstrates that all GBCAs can be found in the brains of rats 24 h after the administration. Slight quantitative differences between the agents seem to exist. In the cerebellum of animals administered gadodiamide, the gadolinium concentration was higher than that of the other GBCAs (Fig. 6).

GBCA kinetics of distribution and excretion from the cerebellum and pons at later time points cannot be described with this study. However, in a recently published mouse study, the brain gadolinium concentration decreased between 3 and 45 days p.i., indicating a persistent GBCA elimination from the brain tissue during this period [37]. Clinical studies have also demonstrated clear differences in brain signal hyperintensity between GBCAs that are associated with their chemical structure [7, 11, 12]. This was confirmed in preclinical investigations by ICP-MS-based brain gadolinium measurements 5 weeks p.i. [26, 28]. The finding of our study, i.e. the fact that all GBCAs independently of their chemical structure initially reach the cerebellum and pons, leads to the hypothesis that the GBCA complex stability plays a role during further elimination from this brain structures. The different chemical structures exhibit different thermodynamic and kinetic complex stabilities [38, 39], apparently allowing for greater release of Gd^3+^ at physiological conditions with linear than with macrocyclic GBCAs [40]. However, to assess the role of complex stability, the chemical species of gadolinium in the brain has to be evaluated. ICP-MS can quantify the gadolinium concentration but cannot distinguish between different forms of gadolinium (e.g. chelated or bound to other chemical species). The development of advanced analytical methods for gadolinium speciation would lead to a more detailed understanding of gadolinium retention in the brain and would shed more light on the role of complex stability.

Although we demonstrated the infiltration and distribution of GBCAs into the CSF, the further GBCA distribution into the brain parenchyma is not conclusive. Assuming that the GBCA in the CSF represents the source of the gadolinium found in the cerebellum and pons, different pathways of distribution might exist. In the classical view, the penetration of substances from the CSF into the brain parenchyma is mediated by diffusion, a process that is much slower than CSF convection and flow. Hence, this process is not very effective and restricted to the upper surface of the brain parenchyma [41]. The perivascular fluid circulation through the Virchow-Robin space surrounding the pial arteries might represent a more efficient route of GBCA transport from the CSF to the brain parenchyma. Recent studies have demonstrated that CSF and interstitial fluid continuously interchange [42]. It is likely that the underlying pathway termed glymphatic system plays an important role in GBCA infiltration from the CSF into the brain tissue [43]. An enhancement of the perivascular space was recently confirmed in humans 4 h after administration of gadoteridol or gadodiamide [44]. However, to date, no connection can be made between the GBCA found in the CSF and the accumulation of gadolinium in the brain.

This study has certain limitations. It was performed on healthy rats without known BBB disorders. This can approach but cannot fully replicate the clinical conditions in patients, especially regarding BBB integrity and function. Gadolinium was quantified in the cerebellum and pons; however, no histological evaluation of these structures was performed. Other limitations arose from the used MRI method. The altered SI levels after MRI scanner servicing do not allow a direct comparison of the two subgroups. In addition, the SI of the heavily T2-weighted FLAIR sequence depends on the T1 and T2 relaxation times of the CSF in a complex manner. Since GBCAs possess different r1 and r2 relaxivites in water [36], a correlation between SI and GBCA concentration is not a constant factor and differs among agents. Thus, FLAIR imaging is more sensitive for GBCAs with a high r1 (e.g. gadobenate dimeglumine) than for GBCA with a lower r1 (e.g. gadoterate meglumine and gadoteridol) [36]. However, gadolinium concentration measurements in the CSF 4.5 h p.i. generally confirm the MRI results.

In summary, this study shows that GBCAs can penetrate from blood into the CSF independent of their chemical structure or physicochemical properties. Only the molecular size seems to be an important parameter as demonstrated by a lower CSF gadolinium concentration after administration of the macromolecular agent gadomer. Dynamic FLAIR MRI demonstrates a kinetic from the inner CSF spaces to the subarachnoid space and suggests a passive distribution and wash-out driven by convection and CSF flow. Twenty-four hours after injection, GBCA clearance from the CSF was almost complete, whereas slightly higher gadolinium concentrations were found in the cerebellum and pons, suggesting delayed excretion from these structures. To date, the mechanism of final distribution from the CSF into the brain and specifically to the DN and the GP could not be evaluated in this experiment and needs further study.
